# The validity of birth and pregnancy histories in rural Bangladesh

**DOI:** 10.1186/s41043-015-0027-8

**Published:** 2015-08-28

**Authors:** Donna Espeut, Stan Becker

**Affiliations:** Department of Population, Family and Reproductive Health, Johns Hopkins University, Baltimore, USA

**Keywords:** Bangladesh, Birth histories, Displacement, Omission, Fertility estimation, Maternity history, Mortality estimation, Pregnancy histories, Validation

## Abstract

**Background:**

Maternity histories provide a means of estimating fertility and mortality from surveys.

**Methods:**

The present analysis compares two types of maternity histories—birth histories and pregnancy histories—in three respects: (1) completeness of live birth and infant death reporting; (2) accuracy of the time placement of live births and infant deaths; and (3) the degree to which reported versus actual total fertility measures differ. The analysis covers a 15-year time span and is based on two data sources from Matlab, Bangladesh: the 1994 Matlab Demographic and Health Survey and, as gold standard, the vital events data from Matlab’s Demographic Surveillance System.

**Results:**

Both histories are near perfect in live-birth completeness; however, pregnancy histories do better in the completeness and time accuracy of deaths during the first year of life.

**Conclusions:**

Birth or pregnancy histories can be used for fertility estimation, but pregnancy histories are advised for estimating infant mortality.

## Background

Population-based surveys such as the Demographic and Health Surveys (DHS) play an important role in generating evidence for decision-making, particularly in countries where population-level data are either scarce or of poor quality. DHS always include a module known as a maternity history. A maternity history is used to gather information that is needed to estimate fertility and child mortality, as well as to identify currently living children in order to assess various aspects of child health and nutrition.

There are two types of maternity histories: birth histories and pregnancy histories. A birth history collects information from mothers on pregnancies that resulted in a live birth, whereas a pregnancy history collects information on both live births and pregnancy losses (i.e., spontaneous abortions, induced abortions, and stillbirths). Each type of history can be either backward or forward in time orientation. A backward history starts with the collection of information on a woman’s most recent birth or pregnancy and goes back in time to her first birth or pregnancy. In contrast, a forward history starts with a woman’s first birth or pregnancy and ends with her most recent. In the World Fertility Survey project carried out between 1974 and 1981, pregnancy histories were collected from representative samples of women in over 40 countries [1]. More recently, the DHS project has used birth histories in over 300 surveys in over 90 developing countries [2]. Both programs have used forward histories.

Given the importance of DHS data in macro-level health planning, it is important to determine the validity of maternity history data. Two types of errors affect the validity of such data: (1) event omission and (2) event displacement in time. Event omission leads to underestimation of demographic indicators derived from the data. Event displacement occurs when the mother inaccurately reports a child’s date of birth, age, or date of death. An event whose timing is incorrectly pushed further back in time than it actually occurred is called “backward displaced,” whereas an event whose timing is incorrectly pushed closer to the date of the interview is called “forward displaced.” Characteristics of the questionnaire, the mother (respondent), the interviewer, and the vital event itself can all contribute to the likelihood of omission or displacement [3].

There have been a number of data-quality assessments involving birth and/or pregnancy histories [3–18]. The DHS program has documented a number of problems associated with fertility and mortality estimation from birth histories. Across 128 surveys between 1985 and 2003, an average of 11 % of births had incomplete information about date of birth, with month of birth the most common missing information [19]. There was also considerable heaping of age at death at 12 months. Another analysis of DHS data showed that missing date of birth information is a more frequent phenomenon among rural than among urban women and in cases where a translator is needed [5]. The DHS program has dealt with the problem of missing or partially recorded date information through standard data editing and imputation procedures.

Previous assessments have also documented reporting problems such as age heaping and digit preference. However, only two of these assessments [3, 8] were actual validation studies; the others were reliability studies or relied on expected patterns to detect problems in data quality. Based on the multi-decade experiences using WFS pregnancy histories and DHS birth histories, it has been argued that birth histories are adequate for estimation of fertility and infant mortality [20]. However, this claim has not been substantiated by research that included both types of maternity histories within the same study. The present study was designed to fill that gap.

## Methods

### Study purpose and hypotheses

In this study, we compare birth and pregnancy histories in terms of the validity of fertility and infant mortality information at the individual level. We test three hypotheses regarding the two questionnaire types—specifically that both types of histories have the same:

1. Completeness of live births, early neonatal deaths, neonatal deaths, and infant deaths
2. Differences between reported and actual “total fertility”
3. Accuracy of the time placement of live births, early neonatal deaths, neonatal deaths, and infant deaths

Testing of the first and third hypotheses is based on individual matching of a woman and her live births, early neonatal deaths, and infant deaths between two data sources described below. The second hypothesis is based on aggregate-level fertility measures.

### Data sources

The analyses involve two data sources from Bangladesh: (1) census and vital event data from the Matlab Demographic Surveillance System (DSS) and (2) birth and pregnancy history data from the 1994 Matlab Demographic and Health Survey (MDHS). Each is now described.

#### The Matlab DSS

Matlab, a rural sub-district in southeastern Bangladesh, is home to a DSS that has been operational since 1966. The International Centre for Diarrhoeal Disease Research, Bangladesh (ICDDR,B) manages the DSS, which covers 149 contiguous villages that are divided into two areas: (1) the Maternal and Child Health Family Planning area (henceforth referred to as the “program area”), where intensive public health interventions have been in existence since 1978 and (2) a comparison area (henceforth referred to as the “non-program area”), where villages are almost exclusively dependent upon government-sponsored programs.

The hallmark of the DSS is longitudinal surveillance of the population. At the time of the fieldwork for this study in 1994, Community Health Workers (CHWs) were visiting households bi-weekly in their assigned areas, inquiring about vital events and changes in family composition (pregnancy outcomes, deaths, marriages, divorces, or migration) and making entries in a notebook. Field supervisors made monthly visits to each household with the CHW and completed event registration forms as indicated: pregnancy outcome, death, marriage, in-migration, out-migration, and divorce [21].

In 1982, ICDDR,B created a DSS Database that consolidates all vital information on each DSS resident. A 10-digit registration number is assigned once to an individual and is permanent. There is a minimum residency of 6 months in the DSS in order to be included in the Database. Although completeness of the DSS data has not been assessed formally, it is a good assumption that vital event ascertainment is near 100 % due to the timely nature of the data collection and quality control. Thus, the DSS is a viable “gold standard” data source for validation of vital event reporting by women in the Matlab population.

#### The 1994 MDHS

The MDHS took place from April to May 1994, immediately after the 1993–1994 Bangladesh Demographic and Health Survey (BDHS) [22]. The rationale for conducting the MDHS was twofold: (1) to ascertain the plausibility of 1993–1994 BDHS fertility estimates (which were much lower than anticipated) and (2) to document the level of validity of birth histories versus pregnancy histories.

MDHS Sample: A stratified random sampling design was used, with each of the 149 DSS villages as a stratum and a fixed sampling fraction of 22/300 = 0.073 within each village in order to yield a desired sample size of approximately 3000 women. With this schema, 3,225 households were selected from DSS villages. Of the households selected, 3,037 were occupied dwellings and had persons present and 3009 had completed household interviews. Of 3,480 women age 15–49 in these households, 3039 had completed interviews (a 97.4 percent response rate).

Also selected were 250 households from 12 neighboring villages just outside of the DSS area. These non-DSS villages were chosen because they were comparable to the adjacent DSS villages in terms of their geographic, economic, social, and religious characteristics. The comparison of the survey data from respondents in the non-DSS villages with data from respondents in the adjacent DSS villages provides insight on the generalizability of the validation results. In particular, we wanted to test for possible biases of survey responses of DSS women since they have been asked about vital events for so long that they may give more accurate responses than would otherwise be the case. Such a bias is referred to as a ‘contamination effect’ below.

Questionnaires: One-half of the MDHS questionnaires contained a standard birth history module (as was used in the 1993–94 BDHS) and the other half contained a pregnancy history module. Both types of maternity history were forward histories and had similar formats, with the exception of three pregnancy history questions on non-live birth pregnancies. (See below.)

Data collection: Thirty-two of the 48 interviewers who were involved in fieldwork for the 1993–94 BDHS were engaged to conduct the interviews in the MDHS. Having the same interviewers in both the BDHS and MDHS effectively controlled for interviewer effects. Interviewers administered a birth history or pregnancy history questionnaire by random assignment within each village, i.e., about half received each type.

An area-level validation study of fertility, infant mortality, and contraceptive prevalence estimates from the MDHS compared to the DSS has been published, noting some discrepancies between MDHS- and DSS-derived estimates [8]. Survey-based TFR estimates were accurate for the 60-month period before the survey (3.18 and 4.40 for Matlab program and non-program areas, respectively) compared to corresponding DSS values for the same period (3.15 and 4.37, respectively). Also, the survey-based estimates of infant mortality were consistent with DSS estimates for the 5-year period prior to the survey. However, survey-based estimates considerably underestimated infant mortality for the 5–9-year period before the survey (MDHS estimates of 71.1 and 84.0 per 1000 live births for program and non-program areas, respectively, compared with corresponding DSS values of 89.6 and 105.0 per 1000 live births). The present study investigates these discrepancies using individual-level data for women who met the inclusion criteria for the validation analysis.

Data processing: The original MDHS data were entered into the Integrated System for Survey Analysis (ISSA, [23]), and standard DHS range and consistency checks were done. Modifications to this original MDHS data file were needed in order to conduct the validation with individual-level matched data from the DSS. Specifically, the Bengali names of mothers and their births (as originally recorded on the paper questionnaires) were appended to the MDHS records. The mother’s full name was the key variable used to match individual births in the MDHS and DSS files, followed by the sex and birth order of each of her births. (Details of the matching are available from the first author.)

For the analysis of possible contamination, first, we compared background characteristics of women in the 12 non-DSS villages with those of women in the 17 adjacent DSS villages, and another comparison was with the characteristics of all women in the DSS sample. Second, we compared levels of three indicators of data quality between the DSS and non-DSS samples. If contamination exists, we would expect data quality to be superior for women in the DSS villages. The three indicators were (1) heaping of women’s ages on ages ending in 0 or 5 (e.g., 15, 20, 25, 30, etc.), (2) the percentage of women who gave a complete birth date (month and year) for all births, and (3) heaping of birth interval lengths on multiples of 12 months.

### Study outcomes

Various pregnancy outcomes (live births, stillbirths, abortions) are recorded in a pregnancy history; therefore, mothers who were interviewed using a pregnancy history questionnaire were first asked: “What was the outcome of the pregnancy (live birth, stillbirth, abortion)?” The following questions further helped to classify the pregnancy outcome based on the mother’s responses: “Did the baby cry or show any sign of life after its birth?” and “How many months did the pregnancy last?”

In both the pregnancy and birth history questionnaires, the interviewer identified deaths by asking the mother the following for each live birth reported: “Is (NAME) still alive?” If the mother reported that the child was not alive, the interviewer then asked: “How old was he/she when he/she died?” Deaths occurring before the second birthday were recorded in months, and deaths occurring within the first month of life were recorded in days. (Deaths at 2 years and above were recorded in years.)

The analysis focuses on four demographic outcomes: (1) live births, (2) early neonatal deaths, (3) neonatal deaths, and (4) infant deaths. We used the following definitions for the analyses:

- *Early neonatal deaths*—live births that die within the first week (0 to 6 days) of life
- *Neonatal deaths*—live births that die within the first month (0 to 29 days) of life
- *Infant deaths*—live births that die within the first year (0 to 11 months) of life

Validation indicators: The match rate, more specifically, the proportion of vital events of a certain type (i.e., live birth, early neonatal death, neonatal death, or infant death) documented in the DSS that were also reported in the MDHS, was the main indicator of the completeness of event reporting. Because the DSS was the “gold standard” data source in the analysis, the denominator was the total number of events identified in the DSS during a given time period.

The accuracy of time placement can obviously be assessed only for matched events. Date of birth was recorded in the MDHS using either the western calendar or the Bengali calendar. (Two Bengali months overlap with one English calendar month.) For each matched live birth, the date of birth was converted to the completed number of months preceding the survey (i.e., “months ago”). Time displacement was defined as the difference between the DSS date of birth/death and the MDHS date of birth/death, with both of these dates converted to “months ago”. Given the slight differences in the calendars, the “correct” time placement was defined as the actual minus reported difference equal to zero or within ±1 month.

Indicators of displacement for live births were the proportion of matched live births with dates reported: (a) too early (i.e., backward displaced by more than a month), (b) correctly (i.e., within ±1 month of the actual date), and (c) too late (i.e., forward displaced more than a month). The mean number of months backward or forward displaced was also calculated. Another indicator was the mean absolute overall error in months. Key indicators pertaining to the time placement of deaths were the proportion of actual infant deaths reported to have occurred after the first year of life in the MDHS; the proportion of actual neonatal deaths reported to have occurred after the first month of life in the MDHS; and the proportion of early neonatal deaths reported to have occurred after the first week of life in the MDHS.

The phenomenon of heaping—or the tendency to report ages or dates ending in certain digits more than others—was explored by calculating the proportion of DSS infant deaths that were heaped on 12 months in the MDHS, and the proportion of DSS neonatal deaths heaped on 1 month in the MDHS. The rationale for examining those two heaping measures was that deaths heaped on 12 months or on 1 month would be excluded from the numerator when calculating infant mortality rates and neonatal mortality rates, respectively.

Reference periods: Individual-level validation indicators are presented for a 15-year reference period (i.e., June 1979 to May 1994). Aggregate-level fertility and mortality measures (as described below) are validated for a 10-year reference period (June 1984 to May 1994) and are presented in a manner comparable to that of standard DHS surveys (i.e., 5-year periods of analysis for mortality rates; 3-year periods for analysis for fertility rates).

This analysis examined both individual-level and aggregate fertility measures. As mentioned previously, our study used strict inclusion criteria (i.e., each woman included in the validation analysis had at least one birth and did not out-migrate during the reference period). Consequently, aggregate-level fertility measures have been calculated for this specific subset of women, not for all women residing in the DSS during the time frame in question. The validity of 3-year (36-month) “total fertility” estimates for this group of women was assessed by first calculating age-specific birth rates (5-year age groups) for the 36-month period before the survey, summing these and then calculating the actual (DSS) minus (MDHS) “total fertility” estimate. We also examined cumulative fertility from age 15 to age 39 for these women. The latter measure was examined because, beyond the most recent 36-month period, it was not possible to calculate age-specific rates for the entire reproductive span (15–49 years) using the MDHS data. (This is due to the classic lexis problem–that women 15–49 at the time of the survey are age 10–44 5 years earlier so information on women 45–49 is missing for that time period, and so on.) Age-specific birth rates could, however, be calculated for the age range 15–39 for each 36-month period within the 10-year study period.

### Statistics

The significance of differences in event coverage and time placement was determined using independent two-sample *z* tests and *t* tests. These statistics were also used to test for differences between birth and pregnancy histories in the actual minus reported differentials for “total fertility”. The significance of differences in event coverage and time placement was also determined using independent two-sample *z* tests and *t* tests. Multivariate logistic regression was used to estimate differences between birth and pregnancy histories in live birth omission while adjusting for other identified determinants of such omission. Children born to the same woman were regarded as being part of the same “cluster.” As a result, the conventional regression assumption of independent observations does not hold so we used regression with generalized estimating equations (GEE) to account for the correlation of events within the same cluster. The analyses were conducted using SPSS and Stata [24, 25].

The following variables were considered for inclusion in the multivariate model:

- *Maternal variables:* age, years of schooling, parity
- *Event variables:* child’s sex, child’s survival status at the time of the interview; number of months the event occurred before the survey (according to DSS data)
- *Interviewer variables:* age, years of schooling, marital status

In the univariate analysis, we used the Wald statistic to determine whether or not a variable would be included in the multivariate model; those with *p* < 0.25 were retained. In the multivariate model, variables that did not significantly (*p* < 0.05) contribute to the model were excluded, and a final model was fit.

## Findings and Discussion

**Fig. 1:**
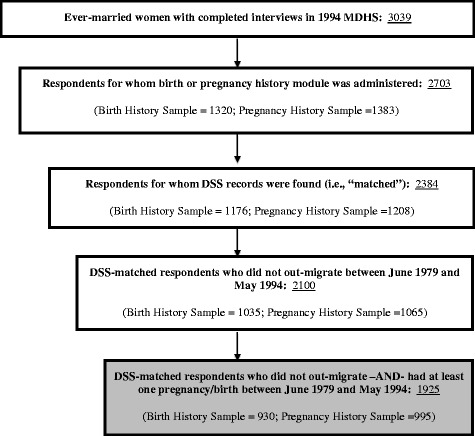
Number of 1994 MDHS respondents and sub-groups of respondents identified as eligible for the present validation analysis

The analysis was based on 1925 MDHS respondents (930 women in the birth history sample; 995 women in the pregnancy history sample) who were matched with their records in the DSS and who had at least one birth or pregnancy during the 15-year period before the 1994 MDHS (i.e., June 1979 to May 1994; Fig. 1). The number with a pregnancy history is slightly larger than the number with a birth history as women with only a non-live pregnancy termination would be included in the pregnancy history analysis sample but not in the birth history analysis sample.

As could be anticipated because of randomization, the birth and pregnancy history samples did not differ significantly in terms of major socio-demographic background factors such as marital status, number of years of schooling, religion, migration status during the study’s reference period, or place of residence (data not shown). Thus, any observed differences in the validation study outcomes were likely due to the type of maternity history, not to those socio-demographic factors.

### Possible contamination

The DSS women were older, had more years of schooling, and were of lower parity than the women in the adjacent, non-DSS villages. However, only the age difference was statistically significant (top panel of Table 1). [We note that the Family Planning and Health Services project was begun in half of the Matlab DSS area in 1977 and led to striking reductions in fertility starting that year [26]. This could explain the slightly older age distribution of women in the DSS area as the number of women aged 15–17 in 1994 would be lower there than elsewhere because of the fertility decline (i.e., 1994–1977 = 17).] The difference between age distributions of women in the DSS villages adjacent to non-DSS villages and women in all DSS villages is likely due to unique characteristics of the former group of villages.

**Table 1:** Background characteristics and indicators of possible contamination for women interviewed in adjacent non-DSS and DSS villages and the women interviewed in all DSS villages, MDHS, 1994

Group of variables and indicator	Place of residence		
	Non-DSS villages	DSS villages	
		DSS villages adjacent to Non-DSS villages	All DSS villages
Number of women	225	292	2703
Background characteristics			
Percent with no schooling	56.4	51.7	54.7
Percent currently married	96.0	94.2	94.5
Percent with age <30 years	55.5	41.5*	48.4
Mean age	30.1	32.7*	31.2
Mean years of schooling	2.10	2.41	2.23
Mean parity	4.31	4.24	3.92
Indicators of possible contamination			
Maternal age heaping (percent of reported ages ending in 0 or 5)	16.9	20.5	19.6
Percent of women who reported complete dates for all births	90.7	97.9*	98.3
Heaping of birth intervals (percent of all intervals 12–48 months reported in multiples of 12)	14 (*n* = 616)	14 (*n* = 764)	15 (*n* = 6498)

*p < 0.05 for the test of equality of means (percentages) in non-DSS and adjacent DSS villages

Regarding the indicators of potential contamination, there was virtually no difference between areas in heaping of birth intervals (bottom panel of Table 1). On the other hand, women in the DSS were more likely to know the complete birth dates of all their births (98 % in DSS villages vs. 91 % in non-DSS villages; *p* < 0.01), which could be attributed to contamination due to the continuous questioning of women about vital events in DSS households. However, surprisingly, women in the DSS were more likely to report an age ending in 0 or 5 than women in the non-DSS villages (21 vs. 17 %, respectively), although the difference was not statistically significant. It is interesting to note that a similar “reverse contamination” was documented as part of the 1980 validation study in Matlab [3]. In summary, though there is some evidence of contamination, we expect the effect to be relatively small since over 90 % of respondents gave complete birth dates for all their births, even in non-DSS villages.

Results are now described for each hypothesis in turn.

### Hypothesis 1: completeness of reporting

Birth and pregnancy histories had virtually identical completeness of live birth reporting at 99 % (Table 2). A child’s survival status was a very important predictor of the completeness of reporting. Among living children, the percentages omitted were 0.2 in the birth history and 0.0 in the pregnancy history. However, the difference between the two histories is more apparent in terms of reporting non-surviving children (Table 2). Among dead children, birth histories had a higher proportion omitted (7.0 % compared to 4.2 % in the pregnancy history sample, *p* = 0.07). Among mothers with no formal education and older mothers (30+ years), statistically significant differences exist between the two types of histories, with birth histories having a higher proportion of omitted live births than pregnancy histories (data not shown).

**Table 2:** Number of events and corresponding completeness rates (%) in the survey

Event type	Questionnaire type					
	Both		Birth history		Pregnancy history	
	Actual no. of events	Completeness (%)	Actual no. of events	Completeness (%)	Actual no. of events	Completeness (%)
Number of women^a^	1925	–	930	–	995	–
Live births	5425	99.2	2666	99.0	2759	99.4
All infant deaths 0–119 months before survey	315	84.1	138	81.9	177	85.9
Infants deaths 0–59 months before survey	151	88.1	69	85.5	82	90.2
Infant deaths 60–119 months before survey	164	80.5	69	78.3	95	82.1
Neonatal deaths	192	82.3	84	81.0	108	83.3
Early neonatal	135	80.7	58	79.3	77	81.8
Late neonatal	57	86.0	26	84.6	31	87.1
Post neonatal	123	87.0	54	83.3	69	89.9
Living children	4718	99.9	2339	99.8	2379	100.0
Dead children*	707	94.5	327	93.0	380	95.8

*Observed difference between birth histories and pregnancy histories were marginally significant (*p* = 0.07) among dead children

^a^Number of women who did not out-migrate between June 1979 and May 1994 and whose last pregnancy occurred during that period

#### Infant deaths

Pregnancy histories performed slightly better than birth histories in terms of the completeness of infant death reporting (Table 2); infant death completeness rates were 86 and 82 %, respectively, though this difference was not statistically significant. For the 315 infant deaths occurring during the 10 years preceding the survey, reporting was more complete for infant deaths during the most recent 5-year period (0–4 years before the survey) than during the preceding 5-year period (5–9 years before the survey), with reporting completeness of 88 and 81 %, respectively. For both reference periods, birth histories performed worse than pregnancy histories, although the differences were not statistically significant. In the birth history sample, completeness was 86 % for infant deaths occurring 0–59 months before the survey and 78 % for infant deaths occurring during the 60–119 months before the survey. Corresponding percentages in the pregnancy history sample were 90 and 82 %, respectively (Table 2).

Among infant deaths, the completeness of death reporting was lower for deaths that occurred earlier during the first year of life. For the 10 years preceding the survey, 83 and 81 % of neonatal deaths were captured by pregnancy and birth histories, respectively. Completeness rates were even lower for early neonatal deaths: 82 % of those deaths were captured by pregnancy histories compared with 79 % by birth histories (Table 2).

From the univariate analysis, the following variables were deemed appropriate (*p* < 0.25) for inclusion in the multiple logistic regression predicting omission: current survival status of the child, mother’s age, parity, interviewer’s years of schooling, and interviewer’s marital status. These variables, along with a variable on questionnaire type (birth history versus pregnancy history) were included in the logistic regression model. Three covariates remained in the final model: child’s survival status, maternal age, and parity (Table 3). After adjusting for these three variables, the odds of live birth omission were 2.0 times greater for birth histories than for pregnancy histories and this was borderline significant (*p* = 0.050).

**Table 3:** Logistic regression estimates (using generalized estimating equations) of the odds (and 95 % confidence interval) of a missed live birth using birth or pregnancy histories, adjusted for survival status of the live birth, maternal age, and parity

Parameter	Adjusted odds ratio	95 percent CI
Questionnaire type*		
Birth history (RC)	1.0	–
Pregnancy history	2.0	(1.0, 3.9)
Current survival status of live birth**		
Alive (RC)	1.0	–
Dead	22.5	(9.7, 52.4)
Maternal age at interview (in years)**	0.8	(0.8, 0.9)
Parity**	1.7	(1.4, 2.0)

Data sources: 1994 Matlab Demographic and Health Survey (MDHS); Matlab Demographic Surveillance System (DSS)

*RC* reference category

*p < 0.10 for the test of whether the adjusted odds ratio differs from 1.0

**p < 0.05 for the test of whether the adjusted odds ratio differs from 1.0

### Hypothesis 2: validity of fertility measures

Differences between actual (DSS) and reported (MDHS) “total fertility” for the 36-month period before the survey were small. Cumulative rates to age 39 were also similar for the periods 3–6 years and 6–9 years before the survey (Table 4). Birth histories and pregnancy histories did not differ significantly in the validity of those aggregate fertility measures. (Note that all of these fertility estimates are higher than those from the area-level analysis by definition because in the present analyses, we excluded women who did not experience a pregnancy or birth between 1979 and 1994 [as noted in Fig. 1]).

**Table 4:** DSS and MDHS estimates of “total fertility” (15–49 years) and “cumulative fertility” (15–39 years) among women sampled for the validation analysis, and the DSS-MDHS difference by the three most-recent 36-month intervals before the survey, according to type of questionnaire

Months before survey	Fertility measure	Questionnaire type					
		Birth history			Pregnancy history		
		DSS estimate	MDHS estimate	Difference	DSS estimate	MDHS estimate	Difference
0–35	Total fertility (15–49)	4.9	4.7	+0.2	5.7	5.3	+0.4
	Cum. fertility (15–39)	4.8	4.6	+0.2	5.5	5.1	+0.4
36–71	Cum. fertility (15–39)	4.7	4.8	−0.1	4.8	4.8	0.0
72–107	Cum. fertility (15–39)	5.3	5.1	+0.2	5.2	5.3	−0.1

Data sources: 1994 Matlab Demographic and Health Survey (MDHS); Matlab Demographic Surveillance System (DSS)

Note: “Total fertility” (15–49) is defined here as five multiplied by the sum of age-specific fertility rates, ages 15–49. “Cumulative fertility” (15–39) is defined as five multiplied by the sum of age-specific fertility rates, ages 15–39. “Total fertility” is in quotes because it was calculated only for the women who met the inclusion criterion for the present validation analysis, i.e., women who had given birth in the DSS within the 15 years before the survey

### Hypothesis 3: accuracy of time placement

Birth and pregnancy histories performed identically in terms of the correct placement of live births (44 % for each, Table 5). Backward displacement and forward displacement were symmetric in the MDHS (29 and 28 %, respectively), with virtually no difference. In addition, there was no statistically significant difference between birth histories and pregnancy histories in the mean absolute number of months displaced, hovering around 15 months.

**Table 5:** Percent of matched live births that are backward displaced, reported correctly, or forward displaced relative to the actual date, mean displacement (in months), and percent displaced according to two covariates, by questionnaire type

Variable and category	Questionnaire type		
	Both types	Birth history	Pregnancy history
Number of matched births (*N*)	5356	2636	2720
Percent displaced			
Backward displaced (i.e. reported too old)	28.5	27.9	29.2
Reported correctly	43.9	43.9	43.9
Forward displaced (i.e., reported too young)	27.6	28.2	26.9
Mean number of months displaced			
Backward displaced	−15.6	−15.2	−16.1
Forward displaced	+15.5	+15.5	+15.4
Percent displaced by characteristic of the event			
Survival status at time of interview			
Alive	53.9	54.0	53.8
Dead	71.7	72.3	71.2
Number of months before survey			
0–59	27.6	26.6	28.7
60–119	63.0	64.0	62.1
120–179	76.1	75.7	76.6

Differentials in displacement of births in time were explored according to the sex of the live birth, survival status at the time of the survey, and number of months before the survey. Birth and pregnancy histories performed similarly with respect to displacement according to each of those characteristics. Overall, female births were more likely to be displaced than male births; however, this differential was not statistically significant (not shown). There was, however, a striking observation in terms of displacement according to survival status (Table 5). The date of birth was displaced for 72 % of matched live births that later died, compared with 54 % of matched live births that were still alive at the time of the survey (*p* < 0.05). There was also a strong and significant relationship between displacement and the number of months since the child was born. Twenty-eight percent of live births that occurred 0–59 months before the survey were displaced, compared with 63 and 76 % of live births occurring 60–119 and 120–179 months before the survey, respectively (*p* < 0.05).

Pregnancy histories have a higher proportion of deaths correctly reported within the age ranges for early neonatal, neonatal, and infant mortality. However, the difference was only significant for infant deaths. Ninety-seven percent of 157 matched infant deaths in the pregnancy history sample were reported within the correct age range, compared with only 90 % of 126 matched infant deaths in the birth history sample (*p* = 0.015). Corresponding estimates for neonatal deaths were 94 and 90 % in the pregnancy history and birth history samples, respectively; for early neonatal deaths, the corresponding values were 93 and 89 %, respectively (Table 6).

**Table 6:** Percent of matched death events reported within the correct age range in the MDHS

Age group of death (in DSS) and measure	Questionnaire type		
	Both types	Birth history	Pregnancy history
Infant deaths			
Number of matched events	283	126	157
Percent reported in correct age range*	93.6	89.7	96.8
Neonatal deaths			
Number of matched events	172	76	96
Percent reported in correct age range	91.9	89.5	93.8
Early neonatal deaths			
Number of matched events	120	52	68
Percent reported in correct age range	90.8	88.5	92.6

*p < 0.05 for the test of equality of proportions between birth and pregnancy histories

Regarding heaping of age at death (on month 12 for infant deaths and month 1 for neonatal deaths), birth histories had a higher percentage of infant deaths heaped on month 12 than did pregnancy histories (4 and 1 %, respectively; *p* = 0.07). The two types of maternity history were virtually identical with respect to the percentage of matched neonatal deaths heaped on month 1 (3.6 vs. 3.7 %) (not shown).

Data quality is a concern in any data collection undertaking, and over the decades, demographers have examined quality of data from various population-based surveys. Most tests of data quality are either reliability tests, tests against some known pattern, or comparisons with one or more other sources. One example is tests of age heaping (e.g., when ages ending in 0 or 5 are over-represented). Very few such tests can actually document the validity of the collected data. Thus, demographic surveillance systems offer a unique opportunity to assess the validity of birth and pregnancy history data collected in the manner of a typical DHS survey, the primary source of national fertility, and child mortality estimates in many less-developed countries.

Our three study hypotheses concerning comparable validity of birth and pregnancy histories were generally supported by the data. The “total fertility” measures calculated after matching cases on an individual level were accurate for both birth and pregnancy histories, and the small differences between the survey and gold standard estimates are fairly consistent with what was observed in a previous area-level validation [8] that also used the 1994 MDHS. Results for aggregate measures in the present analysis differed from the results presented in the earlier analysis by Bairagi et al. [8] because the present analysis includes data from a specific sample of women who met the inclusion criteria for this validation analysis, (i.e., they had a birth recorded in the DSS in the 15 years before the survey and did not out-migrate during that period) while the aggregate measures in the previous analyses were area-level measures with all women from the MDHS (and DSS) included.

When calculating woman years for the age-specific birth rates, the present analysis used the DSS maternal date of birth for women less than 28 years of age, not the reported (MDHS) date of birth. Only younger women born in the DSS area had known date of birth, so we could not assess the effect of misreporting of women’s ages for all women. Our analysis of a possible contamination effect was based on women’s reported ages and dates of birth and suggests that there are some inaccuracies in age and date of birth reporting. However, we expect that inaccuracies of maternal age reporting would only have a minor effect on the results.

Both birth and pregnancy histories were close to perfect in terms of the completeness of live birth reports; completeness rates were 99 %. The regression analysis showed, however, that when adjusting for the survival status of the child, maternal age at the time of interview, and parity—three factors observed to have a strong, statistically significant association with live birth omission in this analysis—the odds of a missed live birth were two times higher if a birth history was used rather than a pregnancy history. Also for infant deaths, use of a birth history led to significantly more of these being reported at 12 months (and thus outside the interval for infant deaths) than was true for the pregnancy history. Therefore, for estimation of infant and child mortality, a pregnancy history is preferred.

Although a pregnancy history is a natural, chronological account of all pregnancies, it will take longer to administer than a birth history because of the additional level of information being asked and recorded. Also, a pregnancy history is more delicate to administer than a birth history since women may not want to discuss pregnancy losses. For example, in contexts where abortion is illegal and/or stigmatized, such pregnancy terminations may be difficult to disclose to an interviewer.

Because no previous individual-level validation studies have included both birth and pregnancy histories within the same study, there is no frame of reference against which our findings can be compared. The previous validation study conducted by Becker and Mahmud [3] in Matlab compared the validity of fertility information collected in backward and forward pregnancy histories. That study also documented completeness rates of about 98 % for live births in both backward and forward pregnancy histories.

There is no significant advantage of one type of maternity history over the other in terms of the time placement of live births. For births that were not correctly placed in time, displacement was symmetric; that is, equal proportions of live births were backward displaced and forward displaced. This finding is contrary to results of previous assessments in which there was a documented greater tendency for backward displacement of live births [3, 27, 28]. The present study does, however, corroborate findings from other assessments in terms of the significantly greater time displacement for births that later died, compared with current survivors [2, 29]. This latter observation might warrant further consideration in contexts with high rates of infant and under-five mortality.

Completeness was lower for deaths earlier during the first year of life. In addition, unlike the comparability of birth and pregnancy histories in terms of fertility estimation, when the priority is on measuring mortality within the first year of life, a pregnancy history is the better instrument to use. Deaths are more likely to be omitted in surveys than are surviving children, and this study shows this pattern with the lowest completeness rates for children who died in the early neonatal period. The poorer performance of both types of histories in capturing deaths that occurred during the first month of life is particularly salient given the increased global attention being paid to neonatal health and survival [30–32].

Regarding generalizability of this study’s findings, a number of factors need to be considered. First, long-term exposure of DSS residents to demographic surveillance and public health interventions could render the DSS population quite different from other populations in terms of the recall and reporting of information in a survey. However, in our analyses, only one of three indicators of such potential “contamination” showed evidence of such an effect—98 % of births had complete date information in the DSS area versus 91 % in the adjacent non-DSS villages. Though significant, this is not a major difference that would render the DSS population unique relative to other populations. As a result, the study’s findings are likely to be applicable to other contexts, at least within rural Bangladesh. It is important to note that validation studies like this can only be done where demographic surveillance has been in place for a number of years.

Migration is another consideration. This analysis excluded all out-migrants from the DSS including those who returned at a later time. This is not customary for standard analyses of fertility and mortality using population-based surveys. Although one may argue that this exclusion introduces a selection bias, 88 % of all MDHS respondents with any DSS records met the criterion of no out-migration from the DSS area during the 15-year reference period for the analysis. (Note that migration between villages within the DSS area did not pose a problem because of the unique identification numbers that are used.) Thus, migration probably does not pose a significant threat to the generalizability of our findings.

It is now possible to conduct validation studies like this one in other sites. In particular, any of the INDEPTH sites with complete and continuous registration of births and deaths for 15 or more years could undertake a similar study [33]. The Navrongo site in Ghana is such an example. We recommend that such studies be done soon after a national DHS survey so the same interviewers can be employed, thus controlling for interviewer effects, which can sometimes be substantial in magnitude. Further validation research conducted in different populations representing different demographic scenarios (e.g., different levels and patterns of fertility, mortality, and migration) can provide additional insights on the best way to collect demographic data in surveys.

The ideal data collection system for estimation of fertility and mortality is a vital registration system, but the cost of such a system is prohibitive for many developing countries. The alternative in many countries is a DHS, which includes a full birth history questionnaire that can produce data to estimate fertility and infant and child mortality. There has been a recent movement toward demographic data collection with faster turnaround than the typical DHS. In this vein, mobile phones are now being used by local enumerators to collect questionnaire data in some countries; this platform has been shown to provide results in a much more timely fashion than a DHS [34].

## Conclusions

Birth and pregnancy histories are comparable in terms of their completeness of live-birth reports. However, because birth histories are associated with a higher degree of age of death heaping at 12 months, resulting in the exclusion of deaths that should be included in infant mortality calculations, a pregnancy history is the preferred tool for estimating infant mortality. The lower completeness of neonatal and early neonatal death reporting in both types of histories is salient given the importance of neonatal survival as a global priority. Our study’s findings are likely to be applicable to other contexts, at least within rural Bangladesh, although validation studies of this nature can only be done where demographic surveillance has been in place for a number of years.
